# Prostate MRI: what to consider when shopping for AI tools

**DOI:** 10.1007/s00330-024-10867-5

**Published:** 2024-06-20

**Authors:** Tobias Penzkofer

**Affiliations:** 1 Department of Radiology, Charité Universitätsmedizin Berlin, Berlin, Germany; 2 Berlin Institute of Health, Berlin, Germany

For at least 40 years, there has been hope that prostate cancer could automatically be diagnosed on imaging. Technologies were initially researched on ultrasound images [1], but only with the advent of both prostate MRI and artificial intelligence in the form of deep learning commercially viable systems became broadly available. This now raises the question of how to choose the right tool for a given setting.

The solution should provide the appropriate feature set to fulfill the tasks needed in your specific setting. There are three tasks that can be performed using AI systems for prostate cancer, and most of them are useful for reporting prostate MRI: (a) segmentation (b) detection, and (c) classification. Segmentation is important to determine the location of the gland, but can also deliver secondary benefits such as automatic prostate specific antigen (PSA) density calculation [2]. Detection refers to the process of finding prostatic lesions, that later can be sorted into levels of suspicion for malignancy in the classification step. Many vendors offer some sort of PI-RADS-like suspicion scores, but in many cases, it will not produce a true PI-RADS-score, as adhering to the PI-RADS classification was designed for a human reader.

In addition, it should be considered if the technical prerequisites for the application of the specific tool are met: firstly, has the tool been developed and validated on the imaging vendor, machine, and protocol that is available at your site; secondly, how much flexibility is there for future changes of the vendor, machine, or protocol, and is there, e.g., external imaging you would like to report on with different hardware than your own. Please note that these changes can impact the performance of the used method. To future-proof your investment—which is not only monetary for license and installation costs—but also in terms of organizational overhead. In the end, you are changing your clinical practice and changing again—or reversing back to conventional reporting, could be at least cumbersome, and potentially expensive. Another factor is the seamless integration into your reading workflow up to the point of biopsy planning [3], which might depend on the interoperability of the system available at your site. The Picture Archiving and Communication System (PACS), the Radiology Information System (RIS), and the Hospital Information System (HIS) are the most relevant in this case, and compatibility should be checked before committing to a new component. Compatibility with the Fast Healthcare Interoperability Resources (FHIR) standard is surely a promising feature for an AI solution [4].

Next to these circumstantial factors, performance is of priority when considering implementing AI into your workflow. The most important factor is probably to deliver the value of the MRI pathway [5]—to reduce unnecessary biopsies in the prebiopsy setting, with a high negative predictive value, and a low amount of detected Gleason Grade Group 1 cancers [6]. On the other hand, in a patient population with prior negative biopsies but continually elevated PSA, the positive predictive value for significant cancer becomes similarly important, to avoid missing relevant cancer diagnoses. The possibility to adapt to such differing operating points might be an important feature in your local setting, depending on your patient population [6].

Cost is another important component. There are different pricing models for AI systems in radiology in general and for prostate MRI specifically. The cost structure can be tied to the number of examinations performed, on the availability of specific features, or both. Initially, it might be interesting to choose a pay-per-case model, which comes usually at a higher cost per individual analysis but mitigates the risk of buying a more expensive tier (e.g., a set of 2000 analyses/year) that could go partially unused. Later, these volume-based models usually offer more competitive prices compared to the pay-per-case models. In any case, it must be considered that these costs are in addition to conventional reporting costs, if not offset by the possibility of a reimbursement for automated or AI-based diagnostics or performance gains in terms of reporting speed and/or accuracy [7].

On top of that, installation and training costs can occur initially. Platform solutions, which host several AI solutions in the same setup, can mitigate installation costs, as in many cases the newly desired software can be chosen from a marketplace. Cloud-based services usually have less hardware footprint and can be set up using minimal requirements, such as virtual appliances that only send images and reintegrate the results into the reporting workflow, thereby omitting the need for costly hardware on-premise.

Regulatory considerations may also play a role when establishing an automated, AI-based, prostate MRI workflow. Especially in the case of cloud-based services, the transmission of patient data needs to be cleared by the site’s data protection authority and data security office. The location of the suppliers’ datacenter can be of importance, especially in regions applying the General Data Protection Regulation (GDPR), where transfer of the data without the patients’ explicit consent is restricted. In cases where additional consent is necessary, the implementation of such a system could quickly become unprofitable. Recently ratified legislation also needs to be considered, namely the medical device regulation and the EU AI Act (other legislatures have similar regulations), where explainable AI [8], or at least the documentation of the decision process, could become a mandatory part of the application of AI and can be requested by the subjects of the data processing. To ensure compliance of your investment, it should be a part of the acquisition process to inquire about the implementation of such regulations by the vendor [9].

In summary, several factors should be considered when shopping for an AI system, especially performance to deliver for your and your patients’ needs, feature set, local technological prerequisites and future developments, integration in your workflow, cost for setup and running the system, as well as regulatory requirements. As more solutions become available, comparative use-case studies will surely be available, either from a neutral standpoint or by the vendors themselves.
